# Gas6 Increases Myelination by Oligodendrocytes and Its Deficiency Delays Recovery following Cuprizone-Induced Demyelination

**DOI:** 10.1371/journal.pone.0017727

**Published:** 2011-03-10

**Authors:** Michele D. Binder, Junhua Xiao, Dennis Kemper, Gerry Z. M. Ma, Simon S. Murray, Trevor J. Kilpatrick

**Affiliations:** 1 Multiple Sclerosis Division, Florey Neuroscience Institutes, University of Melbourne, Parkville, Victoria, Australia; 2 Centre for Neuroscience, University of Melbourne, Parkville, Victoria, Australia; 3 Department of Anatomy and Cell Biology, University of Melbourne, Parkville, Victoria, Australia; University of New South Wales, Australia

## Abstract

Multiple sclerosis (MS) is a complex demyelinating disease of the central nervous system. Current research has shown that at least in some cases, the primary insult in MS could be directed at the oligodendrocyte, and that the earliest immune responses are primarily via innate immune cells. We have identified a family of receptor protein tyrosine kinases, known as the TAM receptors (Tyro3, Axl and Mertk), as potentially important in regulating both the oligodendrocyte and immune responses. We have previously shown that Gas6, a ligand for the TAM receptors, can affect the severity of demyelination in mice, with a loss of signalling via Gas6 leading to decreased oligodendrocyte survival and increased microglial activation during cuprizone-induced demyelination. We hypothesised TAM receptor signalling would also influence the extent of recovery in mice following demyelination. A significant effect of the absence of Gas6 was detected upon remyelination, with a lower level of myelination after 4 weeks of recovery in comparison with wild-type mice. The delay in remyelination was accompanied by a reduction in oligodendrocyte numbers. To understand the molecular mechanisms that drive the observed effects, we also examined the effect of exogenous Gas6 in *in vitro* myelination assays. We found that Gas6 significantly increased myelination in a dose-dependent manner, suggesting that TAM receptor signalling could be directly involved in myelination by oligodendrocytes. The reduced rate of remyelination in the absence of Gas6 could thus result from a lack of Gas6 at a critical time during myelin production after injury. These findings establish Gas6 as an important regulator of both CNS demyelination and remyelination.

## Introduction

Multiple Sclerosis (MS) is a complex demyelinating disease of the central nervous system (CNS). Although the initiating insult in MS remains unknown, it is clear that the pathology of MS involves complex interactions between many systems and cell types, including neurons, glia and both the innate and adaptive immune systems.

The majority of research in MS has focussed on the indisputably important role of adaptive immunity in the disease. However, it has recently been posited that, at least in some cases, the primary insult could be directed at the oligodendrocyte. In a seminal study in 2004, Barnett and Prineas examined newly-forming lesions from MS patients and found that prior to the onset of extensive demyelination, and in the absence of T-cell infiltration, oligodendrocyte death could be detected, generally in association with increased numbers of microglia [1]. This work suggests that oligodendrocyte apoptosis and the innate immune response could have important roles to play in the initial development of an MS lesion. A family of receptor protein tyrosine kinases known as the TAMs, comprised of three closely related proteins (Tyro3, Axl and Mertk), has been shown to be centrally important in the regulation of both oligodendrocyte survival and microglial activation.

The three TAM receptors were identified as a distinct receptor PTK subfamily in 1991 [2]. The extracellular, ligand-binding regions of these receptors each have a defining arrangement of two tandem immunoglobulin-related domains and two fibronectin type III repeats. These domains are followed by a single-pass transmembrane domain, and a catalytically competent, cytoplasmic PTK [3], [4], [5]. Like all other receptor PTKs, the TAMs signal as dimers. They are activated by the binding of two closely-related ligands, Gas6 (Growth Arrest Specific Gene 6) and Protein S (ProS), which also dimerize (reviewed in [6]).

We, and others, have recently shown that TAM signalling can regulate myelination, microglial activation and phagocytosis during both demyelination and remyelination in the CNS. We showed that Gas6 knockout (KO) mice subjected to cuprizone-induced demyelination for three weeks exhibited significant differences in both myelination and microglial activation in comparison with wild-type (WT) mice: in the absence of Gas6, oligodendrocyte survival was compromised, demyelination was worse and there were fewer myelinated axons [7]. In contrast, a study by Hoehn *et al.* of cuprizone challenge to Axl KO mice did not observe an increased loss of oligodendrocytes in comparison with wild-type mice after 6 weeks of cuprizone-challenge [8]. This apparent disparity between the relative effect of Gas6 and Axl deficiency upon oligodendrocyte survival in mice challenged with cuprizone suggests that a loss of signalling through a single receptor (Axl) has a less profound effect than a reduction in signalling through all three receptors, such as in the absence of the ligand Gas6. Alternatively, it could be that either Tyro3 or Mer is the main TAM receptor responsible for transducing the anti-apoptotic effect of Gas6 in this context.

In addition to the studies in animal models of demyelination described above, recent evidence has shown that that TAM receptor signalling is involved in both the etiology and pathogenesis of MS. We recently conducted an association study to identify single nucleotide polymorphisms (SNPs) within genes encoding the TAM receptors and their ligands associated with MS. We identified polymorphisms within the *MERTK* gene associated with the risk of developing MS [9]. Further evidence linking TAM signalling to the pathogenesis of MS also has emerged from studies of human MS lesions, revealing that soluble Axl and Mer proteins, which act as decoy receptors to sequester Gas6, are upregulated in chronic MS lesions, and are negatively correlated with expression of Gas6 [10]. These data suggest that a reduction in protective Gas6 signalling could correlate with extended lesion activity.

Given the known role that Gas6 and the TAM receptors play in regulating myelination, we examined the role of these molecules during recovery from cuprizone-induced demyelination, and in an *in vitro* model of myelination. Here, we demonstrate that the absence of Gas6 delays, but does not ultimately prevent, remyelination following cuprizone-induced demyelination, and that this delay is correlated with a reduction in oligodendrocyte numbers. This effect may be contributed to by a direct effect of Gas6 upon myelination by oligodendrocytes, as we also show that exogenous Gas6 can enhance myelination of dorsal root ganglion (DRG) neurons *in vitro*.

## Materials and Methods

### Animals and reagents

Gas6^−/−^ mice [11] were backcrossed onto the C57Bl6 background as previously described [7]. Sprague-Dawley rats for primary cell culture were obtained from the Animal Resource Centre (Canning Vale, WA, Australia).

All chemical reagents were obtained from Sigma-Aldrich (St Louis, MO) unless otherwise indicated. Recombinant human Gas6 (rhGas6) was a kind gift of Dr Patrick Jones (Berlex Biosciences, Richmond, CA). All cell culture plasticware was purchased from Nunc (Rochester, NY). All cell culture media and reagents were purchased from Invitrogen (Carlsbad, CA) unless otherwise indicated. All secondary antibodies were purchased from Jackson Immunochemicals (West Grove, PA) unless otherwise indicated.

### Ethics Statement

All animal experiments were approved by the Florey Neuroscience Institutes Animal Ethics Committee (Approval #07-063) and conducted according to National Health and Medical Research Council (NH&MRC) guidelines, with all appropriate effort made to minimise animal suffering.

### Purification and culture of rat oligodendrocyte precursor cells (OPCs)

Oligodendrocyte precursor cells were purified from the cortices of P7 Sprague-Dawley rat pups using sequential immunopanning as previously described [12]. Purified cells were cultured in 75 cm^2^ tissue culture flasks coated with poly-D-lysine (PDL) in modified Bottenstein-SATO medium [13] containing N-acetyl-cysteine (60 µg/ml), forskolin (5 µM), penicillin and streptomycin, neurotrophin-3 (NT-3, 5 ng/ml, Peprotech, Rocky Hill, NJ) and platelet-derived growth factor-AA (PDGF-AA, 10 ng/ml, Peprotech, Rocky Hill, NJ).

### Co-culture of rat dorsal root ganglia (DRG) and OPCs

Rat DRG-OPC co-cultures were established based on previously described protocols [14], [15]. Briefly, rat DRG neurons were purified with antimitotic medium in the presence of NGF (100 ng/ml) for 2–3 weeks as described previously [16]. OPCs were seeded onto coverslips containing purified DRG in a small volume at a density of 200,000 OPCs per 22 mm coverslip or 100,000 OPC per well of 4-well chamber slides and incubated overnight to facilitate attachment. DRG-OPC co-cultures were maintained for 14 days in a defined co-culture media containing 50∶50 DMEM∶Neurobasal medium with Sato and B27 supplements, N-acetyl cysteine and D-biotin with or without Gas6 according to the experimental paradigm.

### Analysis of myelination in DRG-OPC co-culture

For analysis of protein levels using western blot, the lysates of DRG-OPCs co-cultures were separated on an SDS-PAGE gel and transferred to a PVDF membrane, as described previously [16]. Membranes were probed with specific antibodies against myelin proteins (2′,3′-cyclic nucleotide 3′-phosphodiesterase [CNPase, Chemicon, Temecula, CA], myelin associated glycoprotein [MAG, Chemicon, Temecula, CA] and myelin basic protein [MBP, Chemicon, Temecula, CA]), then incubated with HRP-conjugated secondary antibodies (Cell Signalling, Danvers, MA). Anti-β-actin antibody (Sigma, St. Louis, CA) was used as a loading control. Blots shown are representative of 3 independent experiments.

For determination of the number of MBP segments, immunocytochemical analysis of myelinating co-cultures was undertaken, as described previously [16]. Briefly, the co-cultures were fixed with 4% paraformaldehyde, then blocked with 20% calf serum in 0.2% Triton X-100 in PBS. Myelin segments were visualized with an anti-MBP antibody (AB980, Chemicon, Temecula, CA), followed by incubation with secondary antibodies, and images were captured by Zeiss confocal microscopy (Carl Zeiss, Inc. Thornwood, NY, USA). For quantitative analysis, 4 random fluorescent images were taken as above, and the number of MBP positive myelin segments in each field was counted.

### Determination of cell death

Co-cultures of OPCs and DRG were established, as described above. Cells were cultured for either 1, 2, 7 or 14 days with appropriate factors depending on the experimental paradigm. Ethidium homodimer-1 (4 mM) and calcein AM (4 mM) were added to each well. Cells were then visualised on an inverted fluorescent microscope (Zeiss, Thornwood, NY) and live (calcein AM positive) and dead (ethidium homodimer-1 positive) cells counted. For all assays 3 wells were counted per condition and assays were repeated at least two times with independent cell isolations. All results are expressed as % live cells±SEM.

### Induction of demyelination/remyelination

Cuprizone mediated demyelination was induced by feeding 8–10 week old mice powdered feed (Barastoc, Pakenham, Victoria, Australia) containing 0.2% cuprizone (w/w: bis-cyclohexanone-oxaldihydrazone) for 5 weeks. Mice were then returned to a normal diet for either 0, 2, 4 or 10 weeks, according to the experimental paradigm. During the 5 week demyelination phase, feed was refreshed each day. Wild-type littermates were used as controls for induction of demyelination in Gas6^−/−^ cohorts. In one cohort (cohort 3), the wild-type, control animals were supplemented with separately bred C57Bl6 mice to increase available numbers. For each cohort an equal mix of male and female Gas6^+/+^ and Gas6^−/−^ mice were subjected to cuprizone challenge. Consistent with previous findings [17], no gender effect was identified. The brains of animals from cohorts 1 and 2 were embedded, cut and analysed in sagittal orientation, whereas those from cohort 3 were embedded, cut and analysed in the coronal orientation.

### Histology

Mice were anaesthetised and perfused intracardially with PBS followed by 4% paraformaldehyde and the brains embedded in paraffin or, for cryosectioning, were equilibrated successively in 12%, 16% and 18% sucrose in PBS for cryoprotection and then frozen on dry ice. Cryostat sections of 15 µm were collected onto chrom-alum coated slides. For coronal analysis, frozen sections were selected from appropriate regions of the brain: rostral sections were as close to Bregma 0.38 as the series of sections would allow; middle from Bregma −0.70; caudal from Bregma −0.94 [18]. Sagittal paraffin sections (10 µm) were selected as close to lateral 0.12 mm as the series of sections would allow [18]. Myelination in cuprizone challenged animals was assessed using luxol fast blue (LFB)-periodic acid Schiff (PAS) reagent using paraffin embedded sections.

For electron microscopic evaluation of myelin, mice were perfused as above and processed for resin embedding. Semi-thin sections (0.5 µm) were cut to evaluate quality and orientation. Representative samples were then chosen and ultra-thin sections (90 nm) cut and images captured using a Siemens Stereoskop Transmission Electron Microscope (Siemens, Munich, Germany) at 3000×. Sections corresponding to the aforementioned Bregma locations were imaged and a region of interest (ROI) randomly selected for quantification by an observer blind to genotype and treatment.

### Quantification and morphometric analysis of axon myelination

Images of the appropriate ROI were imported into Image J64 (US National Institutes of Health, Bethesda). The threshold function of Image J64 was used to outline the myelin within each image, with threshold levels adjusted manually. The number of myelinated axons within each ROI was counted manually. The measure function was used to determine both the internal area of each myelinated axon and the area of the whole axon including myelin wrappings. The diameter of each axon and total fibre diameter was calculated mathematically from the internal area or the whole axon area respectively, as the diameter of a circle of equivalent area [19]. The *g*-ratio for each axon was calculated as a ratio of axon diameter/fibre diameter. Counts of myelinated axons, axon diameter and g-ratios are expressed as mean± SEM.

### Quantification of oligodendrocyte and microglia number

Sections of frozen tissue were prepared as described above. Sections were fixed in 4% paraformaldehyde. Primary antibodies were used at 1/500 and sourced as follows: anti-Olig2 (Millipore, Billerica, MA); anti-IBA1 (Wako Chemicals, Richmond, VA); anti-GFAP (Dako, Carpinteria, CA); anti-PDGFRα (Fitzgerald, Concord, MA); anti-APC(CC1) (EMD Chemicals, Gibbstown. NJ). Appropriate secondary antibodies were used at 1/500 and the final antibody incubation included Hoescht 33342 (1/2000, Invitrogen) to visualise the nuclei of all cells. For cell counts, images were captured from 3 sections for each individual animal using a Carl Zeiss Axioplan microscope at 20× objective. All immunopositive cells in the corpus callosum contained within the imaged region were counted, and the area of the corpus callosum measured using NIH ImageJ64 (US National Institutes of Health, Bethesda). Counts are expressed as number of positive cells/mm^2^± SEM.

### Quantification of LFB

Image analysis of LFB stained sections was performed according to procedures previously described [20]. Briefly, all images from a given experiment were acquired in a single session using the same light intensity and filter settings with the white balance of all images standardised. Images were captured using a Carl Zeiss Axioplan microscope with a 5× objective. For quantification of LFB density, images were divided into equal thirds corresponding to rostral, middle and caudal segments. The area of the corpus callosum was determined using the NIH ImageJ64 (US National Institutes of Health, Bethesda). Density measurements are expressed as raw mean intensity±SEM.

### Statistical analysis

All statistical analysis was performed using GraphPad Prism 5 software (GraphPad Software Inc., La Jolla). Differences between genotypes were compared using Students t-tests. Single-factor, multiple condition experiments were analysed using one-way ANOVA with Bonferroni's post-hoc tests. A *p*-value of less than 0.05 was considered to be statistically significant.

## Results

### Early remyelination is influenced by the absence of Gas6

**Figure 1:**
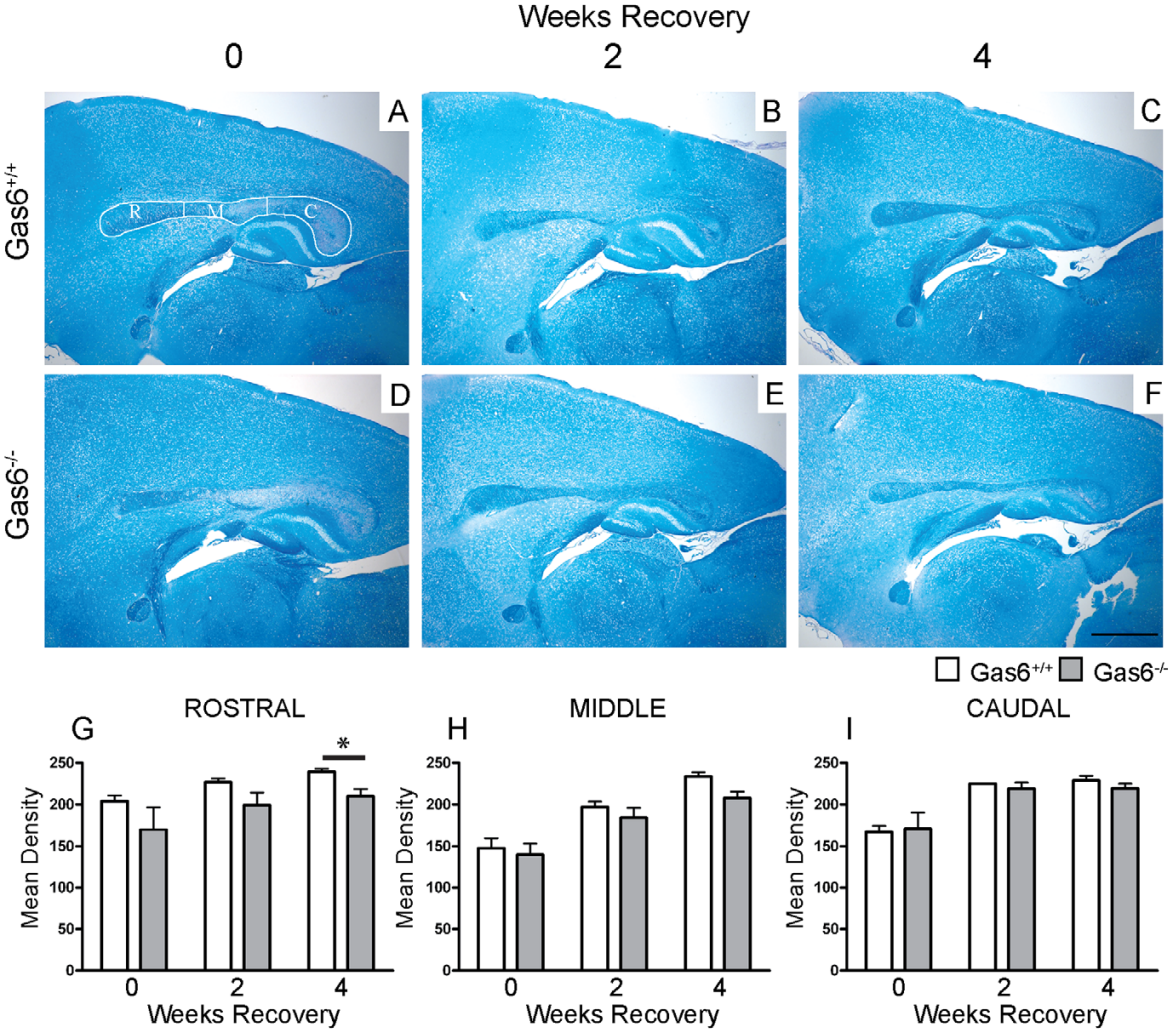
LFB staining demonstrates a reduction in the extent of remyelination in the absence of Gas6. **A–F** Wild-type and Gas6 KO mice were subjected to cuprizone challenge for 5 weeks followed by recovery in the absence of cuprizone for 0, 2 or 4 weeks. **A**. The corpus callosum was divided into three segments for image analysis: rostral (R), Middle (M) and Caudal (C) **G–I** Myelin density was assessed using image analysis as described in the Materials and Methods. **G**. A significant reduction in myelination was observed in the rostral segment of the corpus callosum (p = 0.039) and a strong trend towards reduction was observed in the middle segment (p = 0.052) (**H**). Scale bar = 1 mm.

We initially investigated whether the absence of Gas6 affects the rate or level of remyelination following cuprizone-induced demyelination. Given our previous results showing that demyelination is greater in the absence of Gas6 following 3 weeks of cuprizone challenge [7], we chose a 5 week time-point of cuprizone challenge to provide nadir levels of demyelination in both Gas6^+/+^ and Gas6^−/−^ mice. After 5 weeks of cuprizone challenge, mice were returned to a normal (cuprizone-free) diet for either 0, 2 or 4 weeks. To analyse the extent of remyelination in wild-type and Gas6 knockout mice, the corpus callosum was segmented lengthwise into 3 equal segments as previously described [7]: rostral, middle and caudal (outlined in Fig. 1A), and the level of myelination assessed using LFB staining as described in the Materials and Methods. Representative images of all groups are shown in Figure 1 (A–F). We detected an effect of the loss of Gas6 in the rostral and middle segments, with Gas6^−/−^ mice showing lower myelin density than wild-type mice after 4 weeks of recovery following 5 weeks of cuprizone-challenge, although in the middle segment this was only a trend (rostral p = 0.039, Fig. 1G; middle p = 0.052, Fig. 1H).

**Figure 2:**
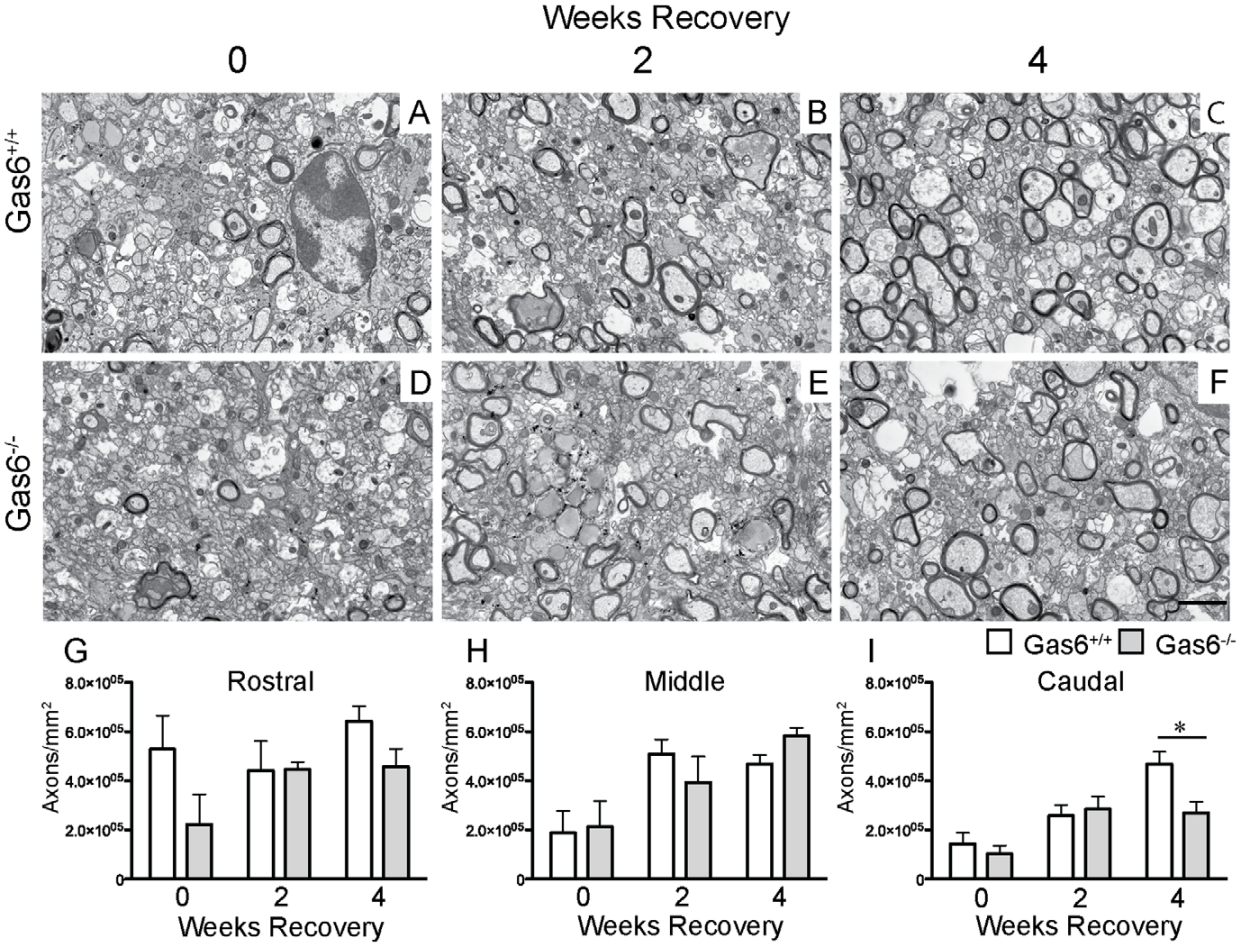
EM analysis demonstrates a reduction in the number of myelinated axons during the course of remyelination in the absence of Gas6. **A–F** Wild-type and Gas6 KO mice were subjected to cuprizone challenge for 5 weeks followed by recovery in the absence of cuprizone for 0, 2 or 4 weeks. The number of myelinated axons per mm^2^ was quantified and shown in **G–I**. A significant reduction in myelination was observed in the caudal (**I**) segments of the corpus callosum in Gas6^−/−^ mice compared with Gas6^+/+^ mice (p = 0.032). Scale bar = 2 µm.

To clarify the changes in remyelination detected using LFB analysis, we next performed an ultrastructural analysis of all segments using electron microscopy. The number of myelinated axons was determined for both Gas6^+/+^ and Gas6^−/−^ mice, as well as the diameter of each myelinated axon and the thickness of the myelin surrounding each axon. Representative images of each group are shown in Figure 2 (A–F). In agreement with the LFB analysis, we detected an effect of the loss of Gas6, with Gas6^−/−^ mice showing fewer myelinated axons than wild-type mice following 4 weeks of recovery. However, in contrast to the LFB analysis this only reached significance in the caudal segment (Fig. 2I; p = 0.032).

In addition to determining the number of myelinated axons in each segment, each myelinated axon was assessed for diameter and myelin thickness (expressed as g-ratio) as described in the Material and Methods, with the data shown in Table 1. No differences between wild-type and Gas6^−/−^ mice were observed in any of the measures, except for a significant increase following 5 weeks of cuprizone challenge in the mean diameter of myelinated axons (Table 1, p = 0.009) and a decrease in the thickness of myelin surrounding the axons in the rostral segment of the corpus callosum of Gas6^−/−^ mice (Table 1, p = 0.007).

**Table 1:** Axon Diameter and *g*-ratios in Gas6^+/+^ and Gas6^−/−^ mice during remyelination.

Weeks Recovery	Genotype	Axon diameter (µm^2^)			*g*-ratio		
		Rostral	Middle	Caudal	Rostral	Middle	Caudal
0	Gas6^+/+^	0.604±0.020	0.781±0.163	0.601±0.041	0.688±0.005	0.703±0.033	0.688±0.014
	Gas6^−/−^	0.718±0.018*	0.645±0.035	0.597±0.060	0.746±0.010*	0.684±0.005	0.648±0.026
2	Gas6^+/+^	0.704±0.039	0.637±0.037	0.588±0.028	0.711±0.010	0.678±0.014	0.651±0.023
	Gas6^−/−^	0.683±0.018	0.657±0.064	0.571±0.062	0.686±0.008	0.715±0.025	0.672±0.017
4	Gas6^+/+^	0.665±0.046	0.626±0.038	0.585±0.011	0.704±0.019	0.677±0.009	0.662±0.013
	Gas6^−/−^	0.722±0.056	0.598±0.027	0.579±0.015	0.705±0.023	0.689±0.012	0.660±0.002

*p<0.05.

Taken together, the analysis of myelination in the absence of Gas6 indicates a significant effect on the remyelination process, with an apparently lower level of myelination after 4 weeks of recovery following 5 weeks of cuprizone challenge, with the maximum effect observed in any parameter being a reduction of approximately 43% in the number of myelinated axons in the caudal segment following 4 weeks of recovery (4.68×10^5^ vs 2.70×10^5^ axons/mm^2^ in Gas6^+/+^ vs Gas6^−/−^ mice respectively; Fig. 2I).

### The absence of Gas6 delays but does not prevent recovery of myelination

**Figure 3:**
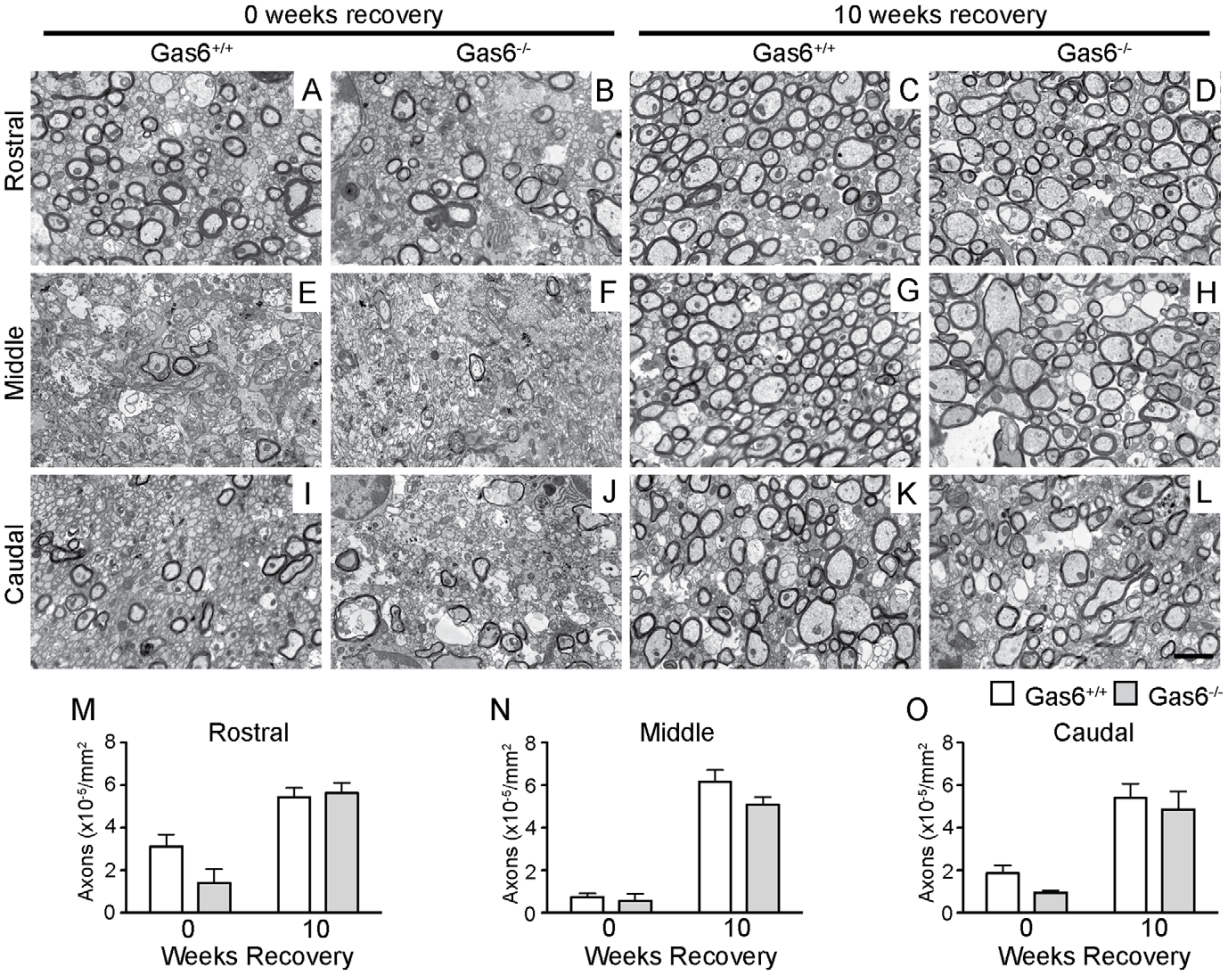
EM analysis demonstrates that, following 10 weeks of recovery, Gas6 KO mice remyelinate to the same extent as Gas6 WT mice. **A–L** Wild-type and Gas6 KO mice were subjected to cuprizone challenge for 5 weeks followed by recovery in the absence of cuprizone for 0 or 10 weeks. The number of myelinated axons/mm^2^ was quantified in the rostral, middle and caudal segments of the corpus callosum (**M–O**). No significant differences were observed between Gas6^−/−^ or Gas6^+/+^ mice at any time point or in segment (p>0.05). Scale bar = 2 µm.

Given the data showing a decrease in myelination following 4 weeks of recovery, we next examined whether this decrease was chronic or if myelin levels recovered to wild-type levels over time. To test this, an independent cohort of mice was subjected to cuprizone challenge for 5 weeks, and then returned to a cuprizone-free diet for either 0 or 10 weeks. As for the previous cohort, ultrastructural analysis was performed using electron microscopy and the number of myelinated axons per mm^2^ quantified. Representative images of each group are shown in Figure 3 (A–L). In contrast to the shorter time-points of recovery, after 10 weeks of recovery following 5 weeks of cuprizone challenge, no significant difference in the number of myelinated axons/mm^2^ was observed between Gas6^−/−^ and wild-type mice in any segment.

In addition, each myelinated axon was assessed for diameter and myelin thickness (Table 2). No difference in either the diameter of myelinated axons or in the thickness of myelin surrounding axons was observed between Gas6^+/+^ and Gas6^−/−^ mice following 10 weeks of remyelination. However, as detected in the previous cohort, small differences were once again observed following 5 weeks of cuprizone challenge, with Gas6−/− mice showing a significant increase in the mean diameter of myelinated axons in the caudal segment (Table 2, p = 0.019), and significantly thinner myelin in the middle segment (Table 2, p = 0.034).

**Table 2:** Axon Diameter and *g*-ratios in Gas6^+/+^ and Gas6^−/−^ mice during long-term remyelination.

Weeks Recovery	Genotype	Axon diameter (µm^2^)			*g*-ratio		
		Rostral	Middle	Caudal	Rostral	Middle	Caudal
0	Gas6^+/+^	0.656±0.030	0.688±0.050	0.511±0.032	0.693±0.017	0.711±0.009	0.687±0.021
	Gas6^−/−^	0.588±0.049	0.764±0.129	0.662±0.039*	0.680±0.012	0.762±0.020*	0.686±0.009
10	Gas6^+/+^	0.642±0.018	0.625±0.022	0.504±0.031	0.699±0.008	0.682±0.011	0.653±0.029
	Gas6^−/−^	0.652±0.016	0.595±0.027	0.571±0.044	0.706±0.010	0.669±0.011	0.659±0.014

*p<0.05.

Taken together, these data indicate that, in contrast to the findings after 4 weeks of recovery following 5 weeks of cuprizone challenge, there is no difference in either the number or size of myelinated axons, or in the thickness of myelin, following 10 weeks recovery.

### Expression of oligodendrocyte markers is reduced following 4 weeks of remyelination

**Figure 4:**
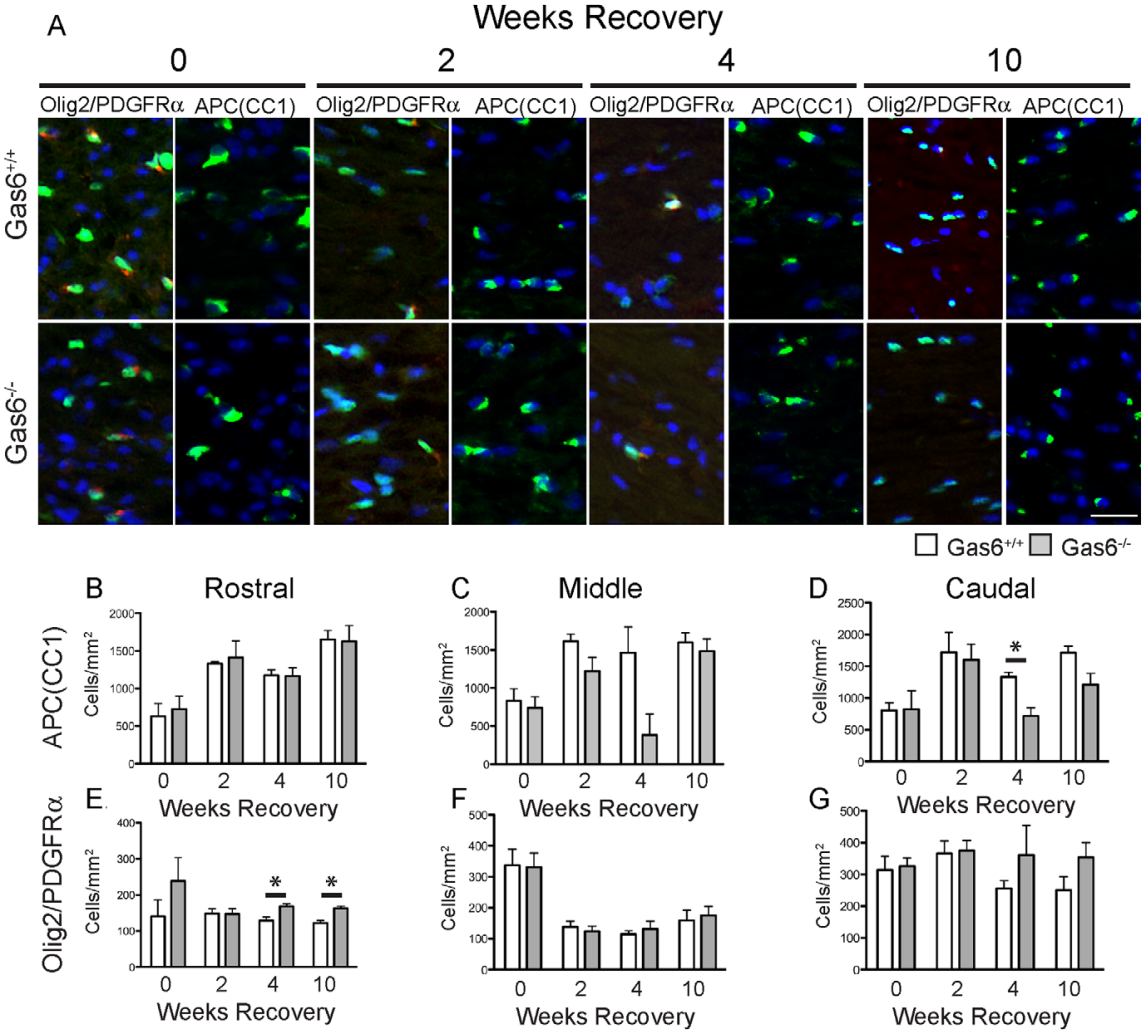
Expression of oligodendrocyte lineage markers are altered in the absence of Gas6 during remyelination. Wild-type and Gas6 KO mice were subjected to cuprizone challenge for 5 weeks followed by recovery in the absence of cuprizone for 0, 2, 4 or 10 weeks. **A**. Representative images of the middle segment of the corpus callosum, showing cells positive for APC(CC1) or double positive for Olig2/PDGFRα. **B–G**. A significant reduction in the number of APC(CC1) positive cells was observed in the caudal segment of the corpus callosum in Gas6^−/−^ mice compared with Gas6^+/+^ mice (p = 0.0070) (**D**). The density of Olig2+/PDGFRα+ OPCs was significantly increased in Gas6^−/−^ mice compared with Gas6^+/+^ mice following both 4 and 10 weeks of remyelination (4 weeks, p = 0.024; 10 weeks, p = 0.003) (**E**). Scale bar = 20 µm.

We next examined whether the delay in the myelination observed in the absence of Gas6 correlated with a reduction in oligodendrocyte numbers. A separate cohort of mice was challenged with cuprizone for 5 weeks and then returned to a cuprizone-free diet for 0, 2, 4 or 10 weeks. Coronal sections were collected as described in the Materials and Methods then stained with anti-APC(CC1) to determine the number of mature oligodendrocytes, with adjacent sections double-stained with anti-Olig2 and anti-PDGFRα to determine the number of oligodendrocyte precursors. The number of positive cells in all 3 segments - rostral, middle and caudal - was determined. Representative images from the middle segment at each time-point are shown in Figure 4(A). We observed a reduction in the number of mature oligodendrocytes in the absence of Gas6 following cuprizone challenge, as measured by expression of APC(CC1), with the caudal segment showing a significant loss of APC(CC1) oligodendrocytes following 4 weeks of remyelination (Fig. 4D, 1331+/−74 cells/mm2 vs 717+/−129 cells/mm^2^, Gas6^+/+^ vs Gas6^−/−^ mice respectively, p = 0.007). Additionally, the middle segment showed a trend towards a reduction in the number of APC(CC1) positive oligodendrocytes following 4 weeks of remyelination (Fig. 4C, p = 0.067). Furthermore, a strong trend towards a reduction in the number of APC(CC1) positive oligodendrocytes was observed in the caudal segment following 10 weeks of recovery (Fig. 4D, p = 0.052).

Taken together, these data indicate that the delay in myelination observed in the absence of Gas6 parallels a reduction in the number of APC(CC1) immunopositive oligodendrocytes. In particular, the only segment to show a significant reduction in APC(CC1) positive oligodendrocytes, the caudal segment following 4 weeks remyelination, was also the sole segment that displayed a significant reduction in the number of myelinated axons at the same time-point.

To assess whether numbers of OPCs were also affected by Gas6 deficiency, the density of PDGFRα/Olig2 double immunopositive cells was determined. The middle and caudal segments showed no apparent differences in OPC numbers at any time-point (Fig. 4H,I). In contrast, the rostral segment of the corpus callosum showed a significant effect, with more OPCs observed in the absence of Gas6 following both 4 weeks of remyelination (Fig. 4E, p = 0.025) and 10 weeks of remyelination (Fig. 4E, p = 0.003).

### Reduction in oligodendrocyte markers is not correlated with an increase in numbers of microglia in the absence of Gas6

**Figure 5:**
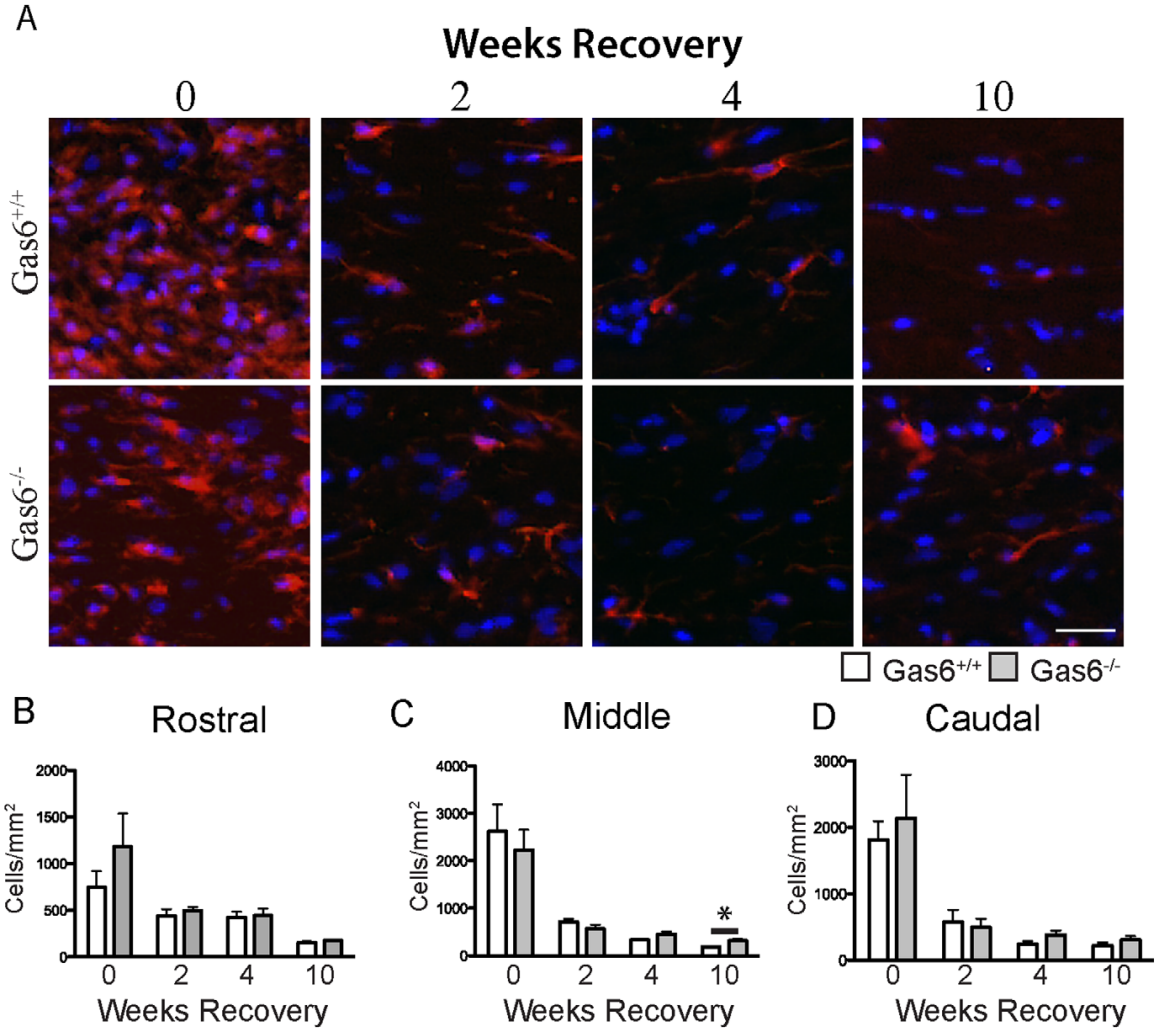
The number of IBA1 positive microglia is minimally altered by the absence of Gas6 during remyelination. Wild-type and Gas6 KO mice were subjected to cuprizone challenge for 5 weeks followed by recovery in the absence of cuprizone for 0, 2, 4 or 10 weeks. **A**. Representative images of the middle segment of the corpus callosum, showing cells positive for IBA1. **B–D** The number of IBA1 positive cells/mm2 was quantified. A significant increase in the number of IBA1-positive microglia was observed in Gas6^−/−^ mice compared with Gas6^+/+^ mice following 10 weeks of remyelination (p = 0.031). Scale bar = 20 µm.

We next wished to determine if the reduction of oligodendrocyte markers in the absence of Gas6 was correlated with an increase in the number of microglia. We therefore assessed the number of IBA1 positive microglia in mice after 5 weeks of cuprizone challenge and following 0, 2, 4 or 10 weeks recovery in the absence of cuprizone. The number of IBA1 positive cells was determined in both Gas6^−/−^ and Gas6^+/+^ mice in all three segments of the corpus callosum - rostral, middle and caudal. Representative images of the middle segment are shown in figure 5A. No significant differences were observed between Gas6^−/−^ and Gas6^+/+^ mice at any time-point examined, with the exception that an increase in the number of IBA1 positive microglia was seen in the middle segment following 10 weeks of remyelination (Fig. 5C, p = 0.013). However, as no difference was observed in the number of APC(CC1) positive oligodendrocytes at this time in this segment, these data indicate that the increased loss of oligodendrocyte markers observed in the absence of Gas6^−/−^ is not directly linked to an increase in the number of microglia.

### Exogenous Gas6 can directly increase myelination of neurons *in vitro*

**Figure 6:**
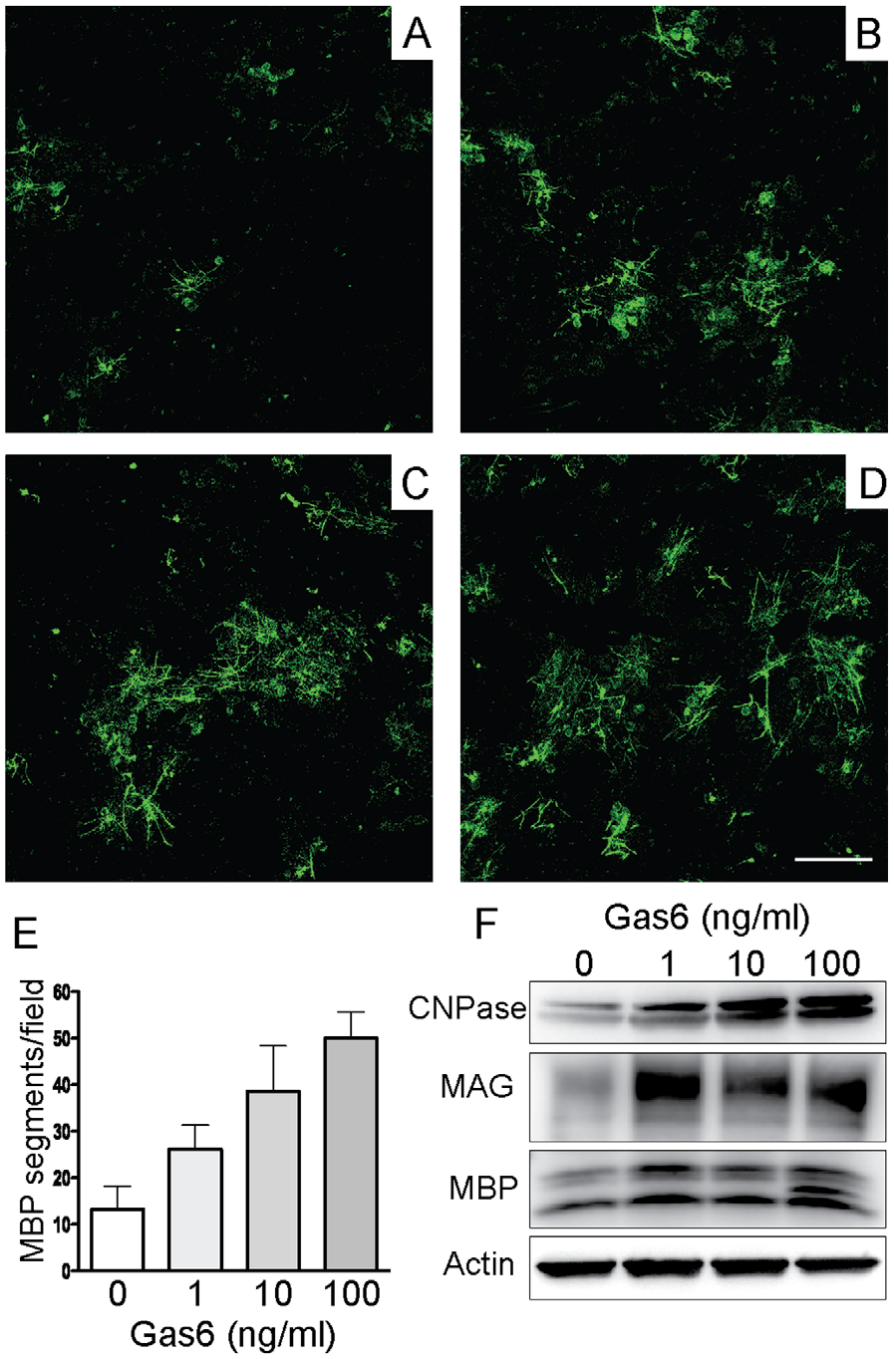
Exogenous Gas6 increases the number of myelinated DRG neurons when co-cultured with oligodendrocytes. **A–D** Oligodendrocytes and DRG neurons were co-cultured as described in the Materials and Methods. Exogenous Gas6 was added to the cultures at either 0 ng/ml (A), 1 ng/ml (B), 10 ng/ml (C) or 100 ng/ml (D) and the number of myelinated segments quantified, with the results shown in (E). Exogenous Gas6 significantly increased the number of myelinated segments in a dose-dependant manner (p<0.0001). **F** Western analysis showing exogenous Gas6 increased the expression of myelin compenents. Scale bar = 100 µm.

We additionally wished to assess if, in addition to affecting numbers of oligodendrocytes, Gas6 could also directly affect the capacity of oligodendrocytes to myelinate axons. In order to assess this, we employed an *in vitro* system, co-culturing OPCs with retinal ganglion cells in the presence or absence of exogenous rhGas6, as described in the Materials and Methods. Upon addition of rhGas6, a dose-dependent significant increase in the number of myelin basic protein (MBP)-positive segments was observed (Figure 6 A–E, p<0.0001). This increase in MBP-positive segments paralleled an increase in the expression of myelin components as determined using Western analysis for CNPase, myelin associated glycoprotein (MAG) and MBP. This increase in myelination did not appear to be a result of improved viability of OPCs in this assay, as no improvement in survival of cells could be detected at 2, 7 or 14 days in the presence of exogenous Gas6 (data not shown). These data indicate that exogenous Gas6 can directly increase the number of myelinated axons in an *in vitro* culture system, suggesting that the delay observed in recovery from cuprizone-challenge in the absence of Gas6 may be partially a result of a deficit in myelination, rather than simply reflecting a loss of oligodendrocytes.

## Discussion

In this study we show that the absence of Gas6 affects the efficiency of remyelination following a demyelinating insult, induced by cuprizone, such that remyelination is delayed, although not ultimately prevented. This delay in remyelination is paralleled by a significant decrease in the number of mature oligodendrocytes. This decrease in oligodendrocytes was specifically observed in the caudal corpus callosum during remyelination, the segment of the corpus callosum most affected by cuprizone. Interestingly, this reduction in oligodendrocyte markers is not correlated with an increase in the number of microglia in the absence of Gas6, suggesting a direct effect of the absence of Gas6 on oligodendrocytes in the remyelination phase. In support of this, we show that exogenous Gas6 can directly enhance the myelination of DRG neurons *in vitro* by oligodendrocytes. Collectively, these data indicate that Gas6 and TAM receptor signalling plays an important role in the process of remyelination following a demyelinating insult, and that this effect could be transduced, at least in part, via a direct effect of Gas6 on myelination by oligodendrocytes.

In demyelinating diseases such as MS, whilst remyelination occurs, it is often inadequate or eventually fails. This failure is thought to result from a variety of factors, including a deficiency of OPCs, or the failure of these OPCs to reach the correct target, or alternatively, failure of differentiation of OPCs resident within lesions (reviewed in [21]). In this study, we show that in the absence of Gas6 there is a significant delay in the remyelination process following cuprizone-induced demyelination. Although remyelination is not ultimately prevented, the decrease in the efficiency of remyelination observed in the absence of Gas6 could lead to an increase in the vulnerability of axons [22], [23]. As the loss of axons has been shown to be closely related to disability in MS (reviewed in [24]), the apparent role of Gas6 in increasing the efficiency of remyelination could be an important factor in preventing or reducing axon loss.

What mediates this delay in remyelination? As indicated above, a number of factors has been postulated to be important in ensuring the efficiency of remyelination, not least of which is the number and localisation of OPCs. In this study, we have shown that whilst there were some changes in the number of PDGFRα/Olig2 positive OPCs in mice in the absence of Gas6 compared with WT mice, specifically in the rostral segment of the corpus callosum, these changes did not clearly correlate with the changes observed in remyelination efficiency. This suggests that the effect of the loss of Gas6 upon remyelination is not upon either the number or recruitment of OPCs to the damaged region. This further suggests that the loss of Gas6 has a direct effect upon either extant or new mature oligodendrocytes during remyelination.

Our examination of mature oligodendrocytes showed that the reduction in myelination observed following 4 weeks of recovery paralleled a significant reduction in the expression of the oligodendrocyte marker APC(CC1). It is not possible to determine from these data whether the loss of marker expression indicates an increase in the death of mature oligodendrocytes or an increase in dysfunction following initial recovery. We and others have previously shown that Gas6 is an important anti-apoptotic signal for oligodendrocytes [7], [25], [26], and it is possible that a reduction in survival signals could lead to the observed effects. However, recent work from Hesse *et al.* [27] indicates that the vast majority of oligodendrocytes undergo apoptosis during the first 3 weeks of cuprizone challenge. Given that differences in the number of mature oligodendrocyte markers between Gas6^−/−^ and Gas6^+/+^ were not observed until 4 weeks post-cuprizone challenge, and that prior to this time the number of both APC(CC1) and Olig2 positive oligodendrocytes was equal between both genotypes, it seems unlikely that the differences observed are the result of cell death, but are more likely to result from either a dysregulation in the expression of these markers and/or in the maturation of these cells.

The mature oligodendrocyte marker APC(CC1) is an antagonist of *β-*catenin and results in a reduction in Wnt signalling [28]. It has previously been shown by Fancy *et al.* (2009) that whilst Wnt-*β*-catenin signalling is upregulated during normal developmental myelination and during remyelination, the timely inhibition of the Wnt-*β-*catenin signalling pathway is required or remyelination is blocked [29]. We observed a significant reduction in APC(CC1) expression in the caudal segment of the corpus callosum and a strong trend for a reduction in APC expression in the middle segment of the corpus callosum after 4 weeks of recovery following cuprizone challenge, the time at which we observed a reduction in myelin density and a reduction in myelinated axons in the absence of Gas6. These findings suggest the intriguing hypothesis that dysregulation in the Wnt-*β*-catenin signalling pathway could be responsible for the reduced efficiency of remyelination observed in the absence of Gas6, a possibility that merits specific interrogation in future experiments.

It is also possible that the observed effects on remyelination could result from other effects of the absence of Gas6 on myelination. These effects could be either direct or indirect. We have previously shown that during demyelination in the absence of Gas6, there was both a loss of oligodendrocytes and a concomitant increase in the number of IBA1-positive oligodendrocytes in the corpus callosum following 3 weeks of cuprizone challenge [7]. However, it was not clear whether the increase in oligodendrocyte loss was a primary effect of the absence of Gas6, or whether it was secondary to the increase in microglial numbers. Hoehn *et al.* (2008) examined a number of time-points in WT and Axl KO cuprizone-mediated demyelination and, in contrast to the findings in Gas6 KO mice, observed a significant decrease in microglia in Axl KO mice after 4 weeks of cuprizone challenge. There was also a delay in phagocytosis and in the removal of myelin debris in these mice [8]. Here we show that the reduction in mature oligodendrocyte markers in Gas6^−/−^ mice occurs in the absence of a differential increase in numbers of IBA1 positive microglia, suggesting a direct effect of the absence of Gas6 on mature oligodendrocytes. Our *in vitro* data showing that exogenous Gas6 can increase the myelination of DRG neurons by OPCs is also strongly suggestive of a direct effect of Gas6 on myelination by oligodendrocytes following a demyelinating challenge. The apparent disparity in the results between the Axl^−/−^ mice and the Gas6^−/−^ mice could reflect the ability of alternate members of the TAM receptor family to compensate for the loss of Axl, whereas the overall reduction in signalling that accompanies the loss of an important ligand could outstrip the ability of, for example, ProS to compensate for this loss.

The results presented in this study indicate an important role for Gas6 and TAM receptor signalling during remyelination, in addition to the known role of this signalling pathway during demyelination. In the absence of Gas6, remyelination is slowed, potentially increasing the length of time to which axons are exposed to damage. Additional work is required to determine the mechanisms by which remyelination efficiency is reduced in the absence of Gas6, in particular whether Gas6 can directly increase myelination by oligodendrocytes *in vivo* as well as *in vitro*.
